# Multi-target Mechanisms and Clinical Evidence for Ganzaoning Granule in Metabolic Dysfunction-Associated Steatotic Liver Disease: A Narrative Review

**DOI:** 10.7759/cureus.110011

**Published:** 2026-05-31

**Authors:** Hetong Zhao

**Affiliations:** 1 Department of Traditional Chinese Medicine, Navy No. 905 Hospital, Naval Medical University, Shanghai, CHN

**Keywords:** ampk signaling pathway, ganzaoning, gut-liver axis, hepatocellular carcinoma, liver fibrosis, metabolic dysfunction-associated steatotic liver disease, network pharmacology, nf-kb, traditional chinese medicine

## Abstract

Metabolic dysfunction-associated steatotic liver disease (MASLD), formerly discussed largely under the non-alcoholic fatty liver disease (NAFLD) framework, is now one of the most common chronic liver diseases worldwide. Its progression from simple steatosis to metabolic dysfunction-associated steatohepatitis (MASH), fibrosis, cirrhosis, and hepatocellular carcinoma (HCC) reflects the interaction of lipid overload, insulin resistance, oxidative stress, immune activation, hepatic stellate cell activation, and oncogenic remodeling. Although resmetirom and glucagon-like peptide-1 receptor agonist-based strategies have recently advanced the treatment landscape for selected patients with MASH and fibrosis, many patients remain outside the indications of current drug therapy, and safe adjunctive strategies across earlier disease stages remain an unmet need. Ganzaoning granule is a traditional Chinese medicine formulation derived from the anti-HCC precursor formula Ganfujian and has been used clinically in China for fatty liver disease and related chronic liver conditions. This narrative review summarizes the available evidence on Ganzaoning, including its phytochemical profile, network pharmacology findings, preclinical studies, and clinical observations in MASLD/NASH populations. Existing studies suggest that Ganzaoning or its related active fractions may modulate lipid metabolism through the AMP-activated protein kinase (AMPK)/peroxisome proliferator-activated receptor gamma coactivator-1 alpha (PGC-1α) axis, attenuate inflammatory signaling involving nuclear factor kappa-light-chain-enhancer of activated B cells (NF-kB) and advanced glycation end-product (AGE)-receptor for advanced glycation end-product (RAGE) pathways, and influence fibrosis- and hepatocarcinogenesis-related molecular processes. However, much of the mechanistic evidence remains preclinical or inferred from network pharmacology, and the clinical evidence is limited by single-center designs, short follow-up, composite endpoints, and limited use of histological or advanced imaging outcomes. We therefore position Ganzaoning as a biologically plausible candidate adjunctive therapy rather than an established MASLD treatment. Future research should prioritize standardized product quality control, pharmacokinetic and pharmacodynamic characterization, herb-drug interaction assessment, and multicenter, double-blind, placebo-controlled trials using accepted MASLD endpoints.

## Introduction and background

The terminology of fatty liver disease has changed substantially in recent years. In 2020, an international expert panel proposed "metabolic-associated fatty liver disease" (MAFLD) to emphasize the metabolic basis of the disorder [1]. In 2023, a multi-society Delphi process introduced "metabolic dysfunction-associated steatotic liver disease" (MASLD) as the preferred umbrella term, with "metabolic dysfunction-associated steatohepatitis" (MASH) replacing non-alcoholic steatohepatitis (NASH) [2]. The 2024 European Association for the Study of the Liver (EASL)-European Association for the Study of Diabetes (EASD)-European Association for the Study of Obesity (EASO) clinical practice guidelines further consolidated the MASLD framework in clinical care [3]. Because MASLD and non-alcoholic fatty liver disease (NAFLD) populations overlap substantially, much of the previous NAFLD/NASH literature remains relevant, but the newer terminology better reflects the cardiometabolic substrate of the disease.

MASLD affects a large and growing proportion of the global adult population. Recent meta-analyses estimate that approximately one-third of adults have NAFLD/MASLD, with prevalence driven by obesity, type 2 diabetes mellitus (T2DM), dyslipidemia, and metabolic syndrome [4,5]. The pathogenesis of MASLD is no longer viewed as a simple two-hit process. Instead, disease progression reflects multiple interacting insults, including insulin resistance, increased de novo lipogenesis, lipotoxicity, mitochondrial dysfunction, oxidative stress, gut-derived inflammatory signaling, hepatic stellate cell (HSC) activation, and immune-mediated injury [6]. These processes can drive the transition from steatosis to MASH, fibrosis, cirrhosis, and hepatocellular carcinoma (HCC), including HCC arising in non-cirrhotic liver [7].

Therapeutic options for MASLD have also begun to change. In March 2024, the United States Food and Drug Administration granted accelerated approval to resmetirom, a selective thyroid hormone receptor-beta agonist, for adults with non-cirrhotic NASH/MASH and moderate-to-advanced fibrosis, in conjunction with diet and exercise [8]. Semaglutide has also shown efficacy for MASH resolution in a large phase 3 trial, supporting the clinical relevance of metabolic therapies in this field [9]. These advances are important, but they apply mainly to defined patient subgroups with biopsy-confirmed or clinically defined MASH and fibrosis. Lifestyle intervention remains central for all patients, and there remains a need for safe, accessible, and mechanistically rational adjunctive approaches, particularly for patients with early-stage disease, multiple metabolic comorbidities, or limited access to newer pharmacotherapies.

Traditional Chinese medicine (TCM) formulas are often developed around multi-component and multi-target principles. This feature may be relevant to MASLD, where metabolic, inflammatory, fibrotic, and oncogenic processes interact rather than occur in isolation [10,11]. Ganzaoning granule is a refined TCM formulation developed from the clinical experience of Professor Ling Changquan at the Naval Medical University. It evolved from Ganfujian, a precursor formula studied mainly in experimental hepatocarcinogenesis and liver fibrosis models. Ganzaoning has been used clinically for fatty liver disease and related liver disorders in China, and later sections of this review summarize its reported bioactive constituents and pharmacological profile. The present review summarizes the current evidence for Ganzaoning in MASLD while explicitly distinguishing network-based hypotheses, preclinical observations, and clinical evidence.

## Review

Methods for this narrative review

This article is a narrative review rather than a systematic review or meta-analysis. The review focused on four evidence domains: (1) MASLD/MASH pathophysiology and current therapeutic standards, (2) the phytochemical and network pharmacology profile of Ganzaoning, (3) preclinical mechanistic studies of Ganzaoning, Ganfujian, or closely related active fractions, and (4) clinical studies of Ganzaoning in fatty liver disease, NASH/MASH, or overlapping chronic liver disease populations.

Relevant literature was identified from PubMed, Web of Science, China National Knowledge Infrastructure (CNKI), Wanfang, and Google Scholar using combinations of the following terms: "Ganzaoning", "Ganfujian", "non-alcoholic fatty liver disease", "NAFLD", "NASH", "MASLD", "MASH", "metabolic dysfunction-associated steatotic liver disease", "AMPK", "NF-kappaB", "hepatic fibrosis", "hepatocellular carcinoma", "traditional Chinese medicine", and "network pharmacology". Priority was given to recent MASLD guidelines and phase 3 therapeutic studies, while older Ganzaoning/Ganfujian studies were retained when they represented direct primary evidence for this formulation lineage. Because the available clinical studies used heterogeneous designs and endpoints and did not provide sufficient uniform data for pooled effect estimation, no meta-analysis was performed. Evidence strength was assessed narratively according to study design, directness of evidence, endpoint validity, sample size, and risk of bias concerns.

Pathophysiological rationale for a multi-target approach

**Figure 1:**
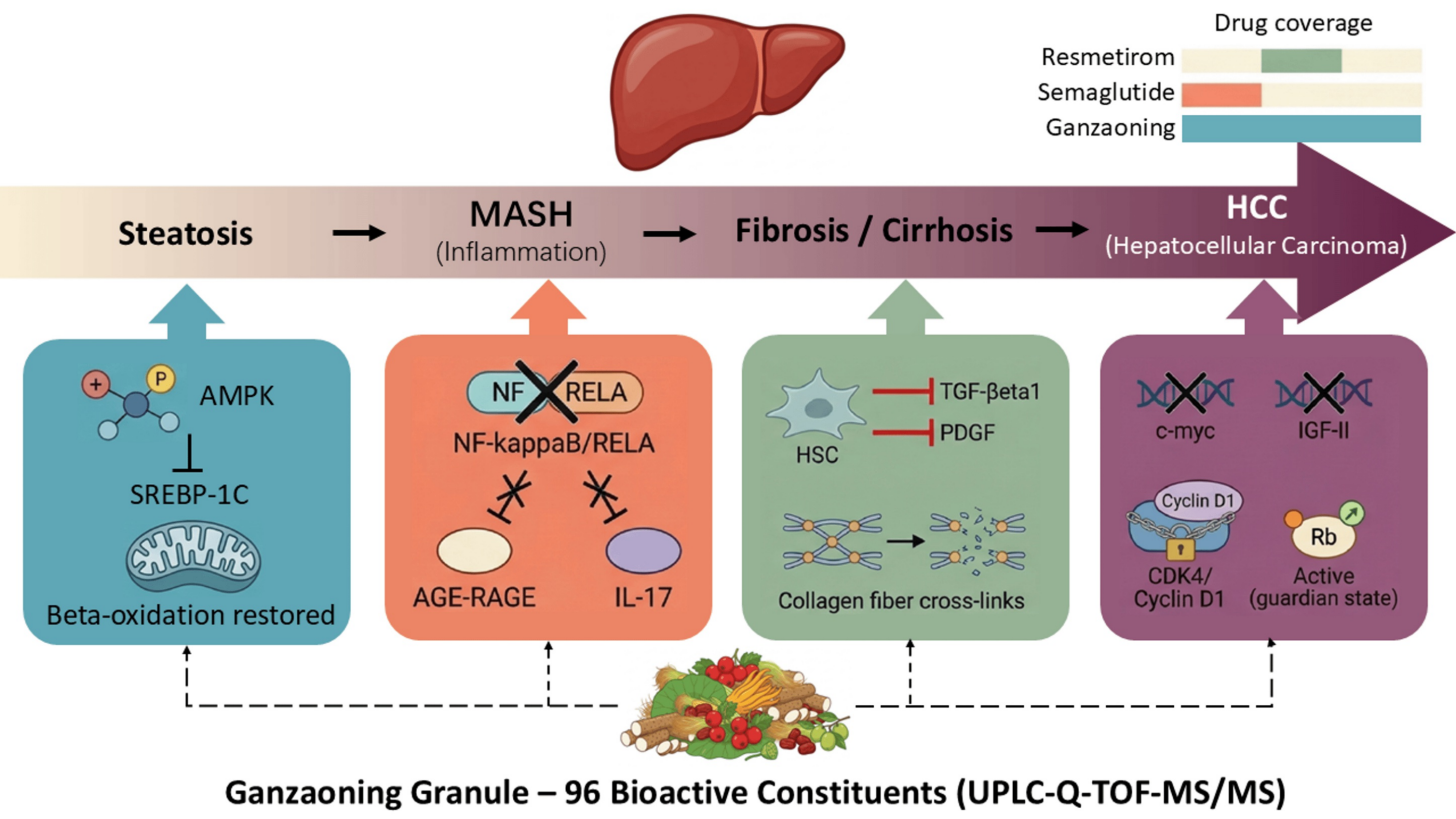
The MASLD disease spectrum and proposed intervention points of Ganzaoning granule MASLD: metabolic dysfunction-associated steatotic liver disease; MASH: metabolic dysfunction-associated steatohepatitis; HCC: hepatocellular carcinoma; AMPK: AMP-activated protein kinase; PGC-1α: peroxisome proliferator-activated receptor gamma coactivator-1 alpha; NF-κB: nuclear factor kappa-light-chain-enhancer of activated B cells; AGE: advanced glycation end-product; RAGE: receptor for advanced glycation end-product; HSC: hepatic stellate cell; SREBP-1c: sterol regulatory element-binding protein 1c; RELA: RELA proto-oncogene, NF-κB subunit; PDGF: platelet-derived growth factor; UPLC-Q-TOF-MS/MS: ultra-performance liquid chromatography-quadrupole time-of-flight tandem mass spectrometry The horizontal axis represents the sequential pathological progression of MASLD from simple steatosis to MASH, hepatic fibrosis, and HCC. Four vertical intervention panels depict the specific molecular targets of Ganzaoning at each disease stage: (1) the AMPK/PGC-1α axis at the steatosis stage, (2) the NF-κB, AGE-RAGE, and IL-17 pathways at the MASH stage, (3) HSC activation interruption at the fibrosis stage, and (4) c-myc, IGF-II, and CDK4/cyclin D1 pathways at the hepatocarcinogenesis-related stage. The image was created by the author using Microsoft PowerPoint (Microsoft Corporation, Redmond, Washington, United States). The mechanistic pathways depicted are synthesized and adapted from the foundational concepts presented in [12-15].

MASLD is a hepatic manifestation of systemic metabolic dysfunction. Its major pathological processes include hepatocellular lipid overload, sterile inflammation, HSC-mediated fibrogenesis, and, in some patients, hepatocarcinogenesis. Figure 1 summarizes the MASLD disease spectrum and the proposed intervention points of Ganzaoning granule across steatosis, MASH-level inflammation, fibrosis, and hepatocarcinogenesis-related pathways. A formulation with multi-pathway activity may therefore be conceptually attractive, but mechanistic breadth should not be interpreted as clinical efficacy unless supported by appropriately designed studies.

Lipid accumulation, insulin resistance, and AMP-activated protein kinase (AMPK) suppression

The earliest hepatic abnormality in MASLD is the accumulation of triglycerides and toxic lipid intermediates such as free fatty acids, diacylglycerols, and ceramides [12]. Insulin resistance increases adipose tissue lipolysis and hepatic de novo lipogenesis, partly through transcriptional regulators such as sterol regulatory element-binding protein 1c (SREBP-1c) and carbohydrate-responsive element-binding protein. AMPK is a central energy sensor that inhibits acetyl-CoA carboxylase and promotes fatty acid oxidation and mitochondrial biogenesis through peroxisome proliferator-activated receptor gamma coactivator-1 alpha (PGC-1α)-related signaling [13,14]. Restoration of AMPK activity is therefore a plausible mechanism for reducing hepatic steatosis, although compound-specific activation and in vivo hepatic exposure require direct validation.

Sterile inflammation and the transition to MASH

Lipotoxic hepatocyte injury can trigger danger-associated molecular patterns and gut-derived pathogen-associated molecular patterns that activate Kupffer cells, Toll-like receptor 4, and the NLRP3 inflammasome. These events promote the production of TNF-α, IL-6, IL-1β, and reactive oxygen species, contributing to hepatocyte ballooning and lobular inflammation [6]. NF-κB, particularly the RELA/p65 subunit, is a central mediator of inflammatory gene transcription. The advanced glycation end-product (AGE)-receptor for advanced glycation end-product (RAGE) pathway may further amplify oxidative stress and inflammation in patients with T2DM or metabolic syndrome [15]. For Ganzaoning, these pathways are best interpreted as mechanistic targets suggested by network pharmacology and preclinical models rather than as clinically proven pathways in MASLD patients.

Fibrogenesis and HSC activation

Persistent inflammatory injury promotes TGF-β1- and platelet-derived growth factor-mediated activation of HSCs. Activated HSCs acquire a myofibroblast-like phenotype and produce extracellular matrix proteins, leading to progressive fibrosis. AMPK activation has been reported to suppress HSC activation and proliferation in experimental systems [13,14]. This creates a plausible link between metabolic and anti-fibrotic effects, but the direct anti-fibrotic efficacy of Ganzaoning in human MASLD remains unproven because existing clinical studies have not used histological fibrosis improvement or magnetic resonance elastography as primary endpoints.

MASLD-associated hepatocarcinogenesis

MASLD-associated HCC may occur without established cirrhosis in a subset of patients [7]. Molecular drivers include metabolic stress, chronic inflammation, DNA damage, altered insulin/insulin-like growth factor (IGF) signaling, and cell-cycle dysregulation. Experimental studies of the precursor formula Ganfujian have reported effects on cell-cycle and HCC-related markers in chemically induced hepatocarcinogenesis models [16,17]. These findings support an anti-hepatocarcinogenesis hypothesis, but they do not establish that Ganzaoning prevents HCC in MASLD patients. The manuscript therefore uses cautious language when discussing HCC-related pathways.

Gut-liver axis: emerging but unconfirmed relevance

Gut microbiota dysbiosis has been implicated in MASLD progression through altered intestinal permeability, portal endotoxemia, bile acid signaling, short-chain fatty acid production, and immune activation [18-22]. Many herbal compounds undergo microbial metabolism, and some TCM formulas have been reported to influence gut microbial composition and bile acid metabolism. For Ganzaoning, however, direct evidence linking treatment-induced microbiome changes to MASLD outcomes remains insufficient. This topic should therefore be framed as a future research direction. Studies using 16S rRNA sequencing, metagenomics, metabolomics, bile acid profiling, and causal transfer experiments could clarify whether microbiota modulation contributes to any observed benefit.

Ganzaoning granule: formulation background and constituents

Ganzaoning granule was refined from Ganfujian, a TCM formula studied in experimental models of liver fibrosis and HCC. The two formulations are described as sharing a related pharmacological core, but the equivalence between Ganfujian-derived mechanistic findings and Ganzaoning-specific clinical effects should be interpreted cautiously unless composition, extraction, dose, and quality control are directly comparable. This distinction is important because TCM formulas can vary by herb source, extraction method, processing, and batch standardization.

Within TCM theory, Ganzaoning is intended to address pathological patterns described as damp-heat, blood stasis, and spleen-qi deficiency. In biomedical terms, these traditional concepts are often mapped onto inflammatory activity, microcirculatory disturbance, metabolic dysregulation, and impaired hepatic repair. Such mappings are useful for hypothesis generation, but they should not substitute for pharmacological validation.

Wang et al. used ultra-performance liquid chromatography-quadrupole time-of-flight tandem mass spectrometry (UPLC-Q-TOF-MS/MS) to characterize the aqueous extract of Ganzaoning and reported 96 detectable constituents [23]. Major compound classes included aporphine alkaloids, flavonoids, terpenoids, phenolic acids, and glycosides. Reported alkaloids included (S)-N-nornuciferine, (R)-N-nornuciferine, O-demethylnuciferine, N-demethylnuciferine, and N-demethylmecambrine. Flavonoid constituents included (-)-epicatechin, a compound with reported antioxidant and AMPK-related activity in other experimental contexts. However, identification in an extract does not by itself establish bioavailability, hepatic exposure, or contribution to clinical efficacy. Future studies should determine which constituents or metabolites reach the liver at pharmacologically active concentrations after oral administration.

**Figure 2:**
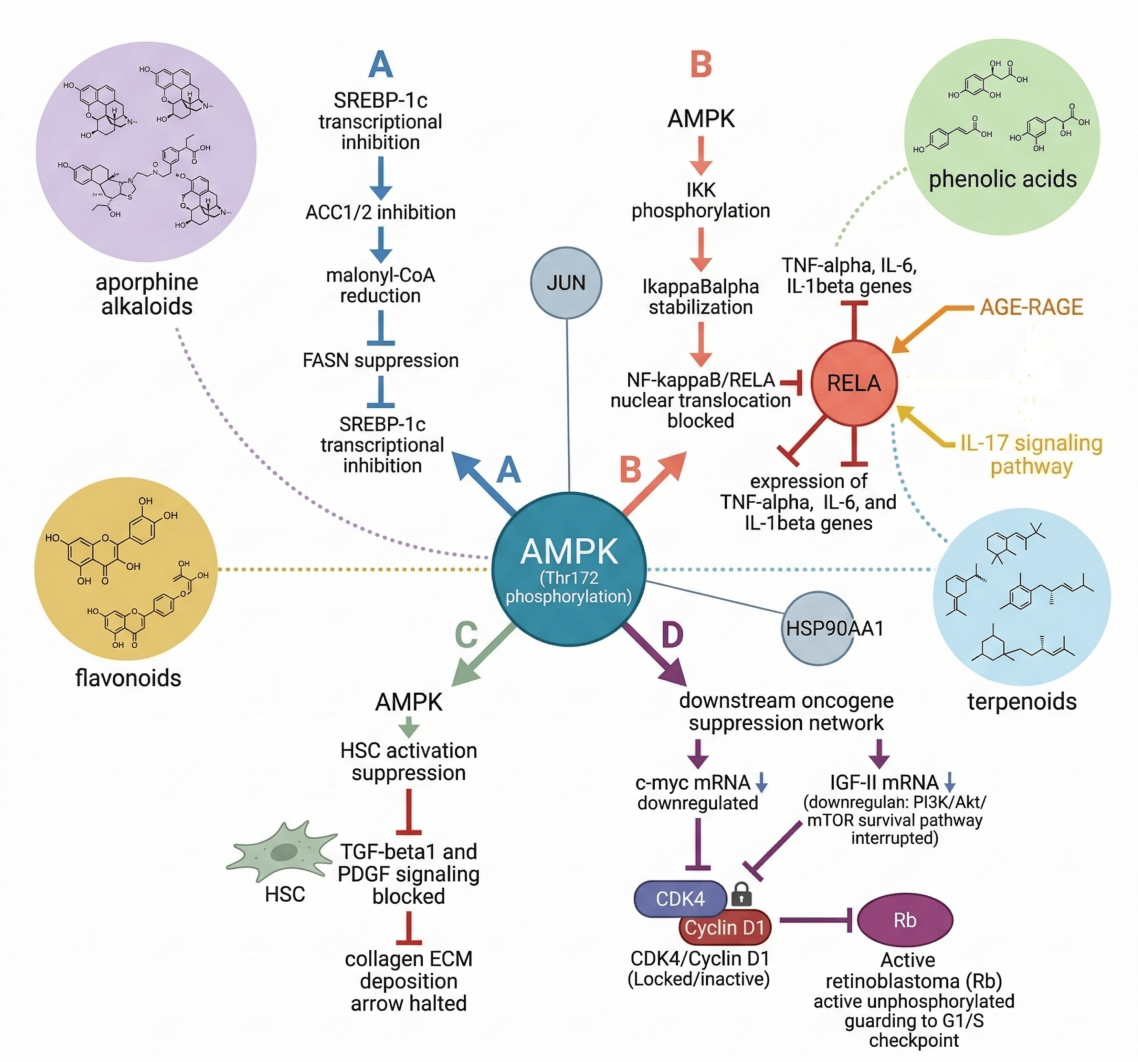
Network pharmacological target landscape and central signaling hub of Ganzaoning in MASLD MASLD: metabolic dysfunction-associated steatotic liver disease; AMPK: AMP-activated protein kinase; SREBP-1c: sterol regulatory element-binding protein 1c; NF-κB: nuclear factor kappa-light-chain-enhancer of activated B cells; HSC: hepatic stellate cell; RELA: RELA proto-oncogene, NF-κB subunit; PDGF: platelet-derived growth factor; ACC1/2: acetyl-CoA carboxylase 1/2; FASN: fatty acid synthase; IKK: IκB kinase; ECM: extracellular matrix The figure presents AMPK as the central molecular hub with primary downstream branches inhibiting lipogenesis (SREBP-1c), attenuating inflammatory gene transcription (NF-κB/RELA), reducing fibrogenesis (HSC inhibition), and modulating downstream oncogene networks (c-myc, IGF-II, and CDK4/cyclin D1). The bioactive constituent classes, including aporphine alkaloids, flavonoids, terpenoids, and phenolic acids, are depicted as feeding into these specific hubs. The image was created by the author using Microsoft PowerPoint (Microsoft Corporation, Redmond, Washington, United States). The network pharmacology mapping data used to conceptualize this figure are derived from Wang et al. [23].

Network pharmacology analysis mapped Ganzaoning constituents to putative liver disease targets, including JUN, RELA, HSP90AA1, and APP [23]. Pathway enrichment suggested the involvement of AMPK, AGE-RAGE, and IL-17 signaling pathways. These results are mechanistically coherent with MASLD biology, particularly lipid metabolism, inflammatory signaling, and immune-fibrotic crosstalk. Figure 2 illustrates this network pharmacology-derived target landscape and the proposed relationship between major constituent classes and MASLD-relevant pathways. Nevertheless, network pharmacology is hypothesis-generating. It should be followed by target engagement studies, dose-response experiments, and in vivo validation rather than being treated as proof of a causal mechanism.

Table 1 summarizes the main constituent classes, proposed molecular relevance, and current evidence level for Ganzaoning.

**Table 1:** Constituent classes, proposed targets, and evidence level for Ganzaoning AMPK: AMP-activated protein kinase; MASLD: metabolic dysfunction-associated steatotic liver disease; NF-κB: nuclear factor kappa-light-chain-enhancer of activated B cells; AGE: advanced glycation end-product; RAGE: receptor for advanced glycation end-product; UPLC-Q-TOF-MS/MS: ultra-performance liquid chromatography-quadrupole time-of-flight tandem mass spectrometry; RELA: RELA proto-oncogene, NF-κB subunit

Constituent or class	Proposed molecular relevance	Current evidence type	Interpretation
Aporphine alkaloids, including nornuciferine-related compounds	Lipid metabolism and inflammatory signaling	UPLC-Q-TOF-MS/MS identification; network prediction	Plausible active class, but hepatic exposure and target engagement need validation
Flavonoids, including (-)-epicatechin	Antioxidant activity; possible AMPK-related effects	Extract profiling; evidence from broader literature	Biologically plausible, but not yet constituent-specific for Ganzaoning in MASLD
Phenolic acids	Oxidative stress and inflammatory modulation	Chemical profiling	Supportive but non-specific
Terpenoids and glycosides	Multi-pathway effects predicted by network analysis	Chemical profiling and computational mapping	Hypothesis-generating
Whole formula or active fractions	AMPK, NF-κB/RELA, AGE-RAGE, IL-17 pathways	Network pharmacology, cell models, animal models	Requires more direct in vivo validation in MASLD models

Preclinical evidence across the MASLD spectrum

In high-fat diet-induced rodent models, Ganzaoning or related active fractions reduced hepatic total cholesterol and triglyceride levels and improved histological lipid accumulation [24]. These findings are consistent with a potential anti-steatotic effect. Mechanistically, AMPK/PGC-1α activation has been proposed as a central pathway, with the downstream inhibition of ACC-mediated lipogenesis and increased mitochondrial fatty acid oxidation [13,14]. However, the available studies do not yet fully define dose-response relationships, active constituents, pharmacokinetics, or whether the same mechanism operates in human MASLD.

In lipopolysaccharide-stimulated RAW264.7 macrophages, active fractions associated with Ganzaoning inhibited nitric oxide production and reduced TNF-α and IL-6 release [23]. These findings support anti-inflammatory potential, particularly through pathways involving NF-κB/RELA. In MASLD, where innate immune activation and metabolic inflammation are central, this mechanism is relevant. Still, cell-based anti-inflammatory activity does not necessarily predict clinical improvement in MASH, and direct evidence in human liver tissue is lacking.

Evidence for anti-fibrotic activity derives mainly from Ganfujian studies in chemically induced liver injury or hepatocarcinogenesis models [25,26]. These studies reported reduced inflammatory infiltration and collagen deposition, including histological evidence by picrosirius red staining. Because Ganfujian is the precursor formula rather than the final Ganzaoning product, these findings should be viewed as supportive lineage evidence rather than direct proof for Ganzaoning. Moreover, diethylnitrosamine (DEN)-induced models do not fully reproduce the metabolic and dietary drivers of human MASLD fibrosis.

Ganfujian studies in DEN-induced rat hepatocarcinogenesis reported lower expression of c-myc, IGF-II, CDK4, and cyclin D1 during liver cancer development [27,28]. These results are relevant because MASLD-related HCC involves proliferative and survival pathways, including insulin/IGF signaling and cell-cycle dysregulation. However, the evidence remains preclinical and model-specific. It supports the investigation of chemopreventive mechanisms but does not justify clinical claims that Ganzaoning prevents HCC.

Clinical evidence and critical appraisal

Clinical studies of Ganzaoning in fatty liver disease and NASH/MASH populations report improvements in liver enzymes, lipid profiles, ultrasound features, and TCM symptom scores [29-32]. The largest reported randomized trial enrolled 300 patients and compared lifestyle intervention alone with lifestyle intervention plus Ganzaoning over three months [30]. The overall response rate was higher in the Ganzaoning group than in the lifestyle-only group, and lipid indices improved. A prospective clinical observation involving 395 patients with NASH reported a total effective rate of 83.29% after six months [29]. Additional work examined Ganzaoning in patients with chronic hepatitis B virus (HBV) infection complicated by fatty liver [31].

These findings are encouraging but should be interpreted cautiously. The main limitations are short follow-up, single-center or regionally limited recruitment, limited blinding, composite "total effective rate" endpoints, ultrasound-based imaging outcomes, and lack of histological endpoints, magnetic resonance imaging (MRI)-proton density fat fraction (PDFF), magnetic resonance elastography, or validated fibrosis biomarkers as primary outcomes. The existing clinical literature therefore supports a preliminary efficacy signal, not definitive therapeutic efficacy. Table 2 provides a structured summary and critical appraisal of the key Ganzaoning/Ganfujian studies cited in this review.

**Table 2:** Summary and critical appraisal of key Ganzaoning/Ganfujian studies AMPK: AMP-activated protein kinase; AGE: advanced glycation end-product; RAGE: receptor for advanced glycation end-product; NF-κB: nuclear factor kappa-light-chain-enhancer of activated B cells; MASLD: metabolic dysfunction-associated steatotic liver disease; NASH: non-alcoholic steatohepatitis; NAFLD: non-alcoholic fatty liver disease; HBV: hepatitis B virus; UPLC-Q-TOF-MS/MS: ultra-performance liquid chromatography-quadrupole time-of-flight tandem mass spectrometry; PK: pharmacokinetics; DEN: diethylnitrosamine

Study	Design or model	Main finding	Main limitation	Evidence interpretation
Wang et al. [23]	UPLC-Q-TOF-MS/MS and network pharmacology	Identified 96 constituents and predicted AMPK, AGE-RAGE, IL-17, and NF-κB-related targets	Computational target prediction; limited in vivo target validation	Useful for hypothesis generation
Zhang et al. [24]	High-fat diet rodent model	Reduced hepatic lipids and improved steatosis-related changes	Animal model; limited PK and constituent-specific data	Supports anti-steatotic potential
Qian and colleagues [25-28]	DEN-induced fibrosis/hepatocarcinogenesis models using Ganfujian	Reduced fibrosis/collagen deposition and modulated oncogene/cell-cycle markers	Precursor formula; chemically induced model; limited MASLD specificity	Supportive but indirect for Ganzaoning in MASLD
Zhang et al. [29]	Prospective clinical observation in NASH	Reported symptomatic, biochemical, and imaging improvement	Non-randomized or limited control information; composite endpoint	Preliminary clinical signal
Lu et al. [30]	Randomized clinical trial in NAFLD	Higher response rate with Ganzaoning plus lifestyle than lifestyle alone	Short duration; likely limited blinding; no histology or advanced imaging	Most direct clinical evidence, but not definitive
Yu [31] and Peng [32]	Dissertation-based clinical and experimental studies	Suggested benefit in fatty liver and HBV overlap settings	Limited accessibility and independent replication	Supportive background evidence

Safety, tolerability, and herb-drug interaction considerations

Reported clinical studies describe a low incidence of adverse events, mainly mild gastrointestinal symptoms that resolved without treatment discontinuation [30]. No consistent signal of hepatotoxicity, nephrotoxicity, hematological toxicity, or cardiovascular toxicity has been reported in the available studies. However, the absence of a strong safety signal in short-term studies should not be interpreted as comprehensive safety confirmation. Larger trials with standardized adverse event reporting, laboratory monitoring, and longer follow-up are needed.

Potential herb-drug interactions deserve particular attention in MASLD because patients often receive statins, antidiabetic drugs, antihypertensives, antiplatelet agents, anticoagulants, and antiviral therapy for HBV when relevant. Future studies should evaluate whether Ganzaoning constituents or metabolites affect cytochrome P450 enzymes, UDP-glucuronosyltransferases, P-glycoprotein, organic anion transporters, or bile acid transporters. Until such data are available, Ganzaoning should be used cautiously in patients receiving polypharmacy, especially drugs with narrow therapeutic windows.

Comparison with current MASLD therapies

Ganzaoning should not be positioned as a replacement for guideline-based MASLD care. Lifestyle intervention remains the foundation of management, including weight reduction, dietary modification, physical activity, and cardiometabolic risk control [3]. Resmetirom is now an approved pharmacological option for selected adults with non-cirrhotic NASH/MASH and moderate-to-advanced fibrosis [8]. Semaglutide and related incretin-based therapies are particularly relevant for patients with obesity and T2DM and have shown MASH-related benefit [9]. In this context, Ganzaoning is best viewed as a candidate adjunctive approach requiring validation rather than as a competitor to approved therapies. Table 3 places Ganzaoning alongside selected current MASLD management options to clarify differences in target population, evidence level, strengths, and limitations.

**Table 3:** Contextual comparison of Ganzaoning with selected MASLD management options MASLD: metabolic dysfunction-associated steatotic liver disease; MASH: metabolic dysfunction-associated steatohepatitis; NASH: non-alcoholic steatohepatitis; FDA: Food and Drug Administration; GLP-1: glucagon-like peptide-1; T2DM: type 2 diabetes mellitus; PK: pharmacokinetics; QC: quality control

Intervention	Main target population	Evidence level	Main strengths	Main limitations
Lifestyle intervention	Broad MASLD population	Guideline-supported	Addresses root cardiometabolic drivers; low cost	Long-term adherence is difficult
Resmetirom	Non-cirrhotic MASH/NASH with F2-F3 fibrosis	Phase 3 trial and FDA accelerated approval	Histological endpoints; regulatory pathway established	Restricted indication; long-term outcomes still under study
Semaglutide/GLP-1 receptor agonist strategy	MASH with metabolic comorbidity, especially obesity/T2DM	Phase 3 evidence for MASH resolution	Weight loss and metabolic benefit	Fibrosis benefit and long-term liver outcomes require continued study
Ganzaoning granule	Candidate adjunctive option for fatty liver/MASLD populations	Preclinical studies and limited clinical studies	Multi-target plausibility; short-term tolerability signal	No large blinded multicenter trials; limited PK, QC, and interaction data

Formal cost-effectiveness evidence for Ganzaoning in MASLD has not been established. Any future health-economic comparison should include product standardization, monitoring costs, access, adherence, and clinical endpoint validity.

Pharmacokinetics, quality control, and regulatory requirements

A major barrier to international acceptance of Ganzaoning is the limited pharmacokinetic and quality standardization evidence. UPLC-Q-TOF-MS/MS has identified many extract constituents, but the clinically relevant active species are likely to include absorbed parent compounds, phase 1/2 metabolites, and microbiota-derived metabolites. Future studies should define oral bioavailability, plasma exposure, hepatic tissue distribution, metabolic pathways, and pharmacokinetic-pharmacodynamic relationships for priority marker compounds.

Quality control is equally important. International development would require clear botanical authentication, standardized extraction, validated marker compound assays, batch-to-batch reproducibility, stability testing, contaminant testing, and Good Manufacturing Practices (GMP)-compliant manufacturing. Because TCM formulas are complex mixtures, reliance on a single marker compound may be insufficient. A multi-marker quality specification linked to biological activity would be more informative.

Regulatory development would also require alignment between the intended clinical claim and the evidence package. A claim of symptom improvement, liver enzyme improvement, MASH resolution, fibrosis improvement, or HCC prevention would each require different endpoints, follow-up durations, and safety monitoring. For MASLD/MASH, future trials should consider accepted endpoints such as MRI-PDFF reduction, histological MASH resolution without fibrosis worsening, fibrosis improvement without MASH worsening, elastography-based fibrosis assessment, and long-term liver-related outcomes.

Limitations of the current evidence base

The current evidence base has several limitations. First, many mechanistic claims are derived from network pharmacology or non-human models, and causal target engagement in human MASLD has not been demonstrated. Second, several preclinical studies involve Ganfujian rather than Ganzaoning, which creates uncertainty about formula equivalence. Third, clinical studies are limited by short duration, incomplete blinding information, composite response endpoints, and lack of histological or advanced imaging endpoints. Fourth, TCM formulation heterogeneity, extraction variability, and batch standardization remain underreported. Fifth, pharmacokinetic, pharmacodynamic, herb-drug interaction, and long-term safety data are insufficient. These limitations do not negate the rationale for further study, but they require a more cautious interpretation of efficacy and mechanism.

Future research priorities

Future work should proceed in a stepwise manner. First, Ganzaoning requires standardized chemical and biological quality control, including validated multi-marker assays and batch consistency testing. Second, pharmacokinetic studies should identify bioavailable parent compounds and metabolites and determine whether they reach the liver at concentrations compatible with the proposed mechanisms. Third, preclinical MASLD studies should use diet- and metabolism-based models that better reflect human disease, with target engagement assays for AMPK, NF-κB, HSC activation, and relevant lipid pathways. Fourth, herb-drug interaction studies should be conducted before broad use in MASLD patients with polypharmacy. Fifth, clinical development should prioritize multicenter, double-blind, placebo-controlled trials with adequate duration and internationally accepted endpoints, including MRI-PDFF, elastography, histology, and validated biomarkers where appropriate. These priorities correspond to the main evidence gaps identified in Tables 1-3.

## Conclusions

Ganzaoning granule is a TCM-derived candidate adjunctive therapy with a biologically plausible multi-target profile for MASLD. Available phytochemical, network pharmacology, cell-based, animal, and early clinical studies suggest potential effects on hepatic steatosis, inflammation, fibrosis-related processes, and hepatocarcinogenesis-associated pathways. However, the strength of evidence remains preliminary. Current data support further investigation rather than definitive claims of clinical efficacy, fibrosis reversal, or HCC prevention. The most important next steps are product standardization, pharmacokinetic and interaction studies, direct target validation, and multicenter randomized trials using accepted MASLD endpoints. With these data, the role of Ganzaoning in integrative MASLD management could be evaluated more rigorously and compared more fairly with established lifestyle and pharmacological therapies.
